# Intensity Modulated Photothermal Measurements of NO_2_ with a Compact Fiber-Coupled Fabry–Pérot Interferometer

**DOI:** 10.3390/s19153341

**Published:** 2019-07-30

**Authors:** Philipp Breitegger, Benjamin Lang, Alexander Bergmann

**Affiliations:** Institute of Electronic Sensor Systems, Graz University of Technology, Graz 8010, Austria

**Keywords:** nitrogen dioxide, photothermal interferometry, gas sensor, optical microphone

## Abstract

Sensors for the reliable measurement of nitrogen dioxide concentrations are of high interest due the adverse health effects of this pollutant. This work employs photothermal spectroscopy to measure nitrogen dioxide concentrations at the parts per billion level. Absorption induced temperature changes are detected by means of a fiber-coupled Fabry–Pérot interferometer. The small size of the interferometer enables small detection volumes, paving the way for miniaturized sensing concepts as well as fast response times, demonstrated down to 3 s. A normalized noise equivalent absorption of $7.5\times 10^{-8}$ cm^−1^W/$\sqrt{\mathrm{Hz}}$ is achieved. Additionally, due to the rigid structure of the interferometer, the sensitivity to mechanical vibrations is shown to be minor.

## 1. Introduction

Due to the adverse health effects of NO_2_ [1], monitoring ambient NO_2_ concentrations as well as NO_2_ emissions from vehicles is of interest for citizens, researchers, and legislative purposes [2,3,4,5]. WHO recommends an hourly mean of 200 μg/m${}^{-3}$ (106.4 ppb) and an annual mean of 40 μg/m${}^{-3}$ (21.3 ppb) not to be exceeded [1]. A variety of sensing principles exist for the sensing of NO_2_. For example, electrochemical and metal oxide sensors are low-cost, but lack sensitivity, selectivity, and long-term stability [6]. On the other hand, chemiluminescence detectors are expensive and large in size, but offer reliable measurements and are commonly used to measure NO_2_ concentrations for legislative purposes [5]. Further, optical sensors exist, which provide high spectral selectivity by choosing a light source that matches one or more absorption bands of NO_2_. Among those are photoacoustic and photothermal sensing concepts.

Photoacoustic spectroscopy uses intensity or wavelength modulated light sources, which match one ore more absorption bands of the gas of interest, exciting transitions into higher molecular energy levels. Subsequent collisional deactivation leads to the production of a fast decaying thermal and a propagating, slowly decaying acoustic wave [7]. The detection of the acoustic wave, usually after acoustically resonant amplification, is utilized in photoacoustic spectroscopy [8,9,10] and quartz-enhanced photoacoustic spectroscopy [11,12,13,14].

This work focuses on an interferometric detection scheme, where the temperature change is measured by a refractive index change, induced by the absorption-based heating. As an excitation source, we use an intensity modulated 450 nm laser. The generated refractive index change is measured by means of a fiber-coupled Fabry–Pérot interferometer as the sensing element. Previous publications have demonstrated noise equivalent absorptions of $1.3\times 10^{-7}$ cm^−1^W/$\sqrt{\mathrm{Hz}}$ with intensity modulation for NO_2_ (equal to 700 ppb for 30 mW average laser power) [15] and $1.8\times 10^{-6}$ cm^−1^W/$\sqrt{\mathrm{Hz}}$ [16] or $7.5\times 10^{-9}$ cm^−1^W/$\sqrt{\mathrm{Hz}}$ [17], with wavelength modulation for detection of SO${}_{2}$. Similar normalized noise equivalent absorptions (NNEAs) can be achieved with microstructured hollow-core fibers in combination with photothermal interferometry [18,19]. The response of hollow-core fiber gas sensors, however, is diffusion limited and response times for long, high-sensitivity fibers are usually limited to several tens of seconds [18,20]. Exceptions with response times down to 3 s and detection limits of $7.4\times 10^{-5}$ cm^−1^ for methane have been reported for short fibers [18,21], but lacking statements about the laser power coupled into the fiber prohibit a comparison to other methods.

In this work, we demonstrate sensing of NO_2_ by photothermal interferometry, utilizing a membrane-free optical microphone as interferometer. The 1σ detection limit for 1 s integration time is 348 ppb, equal to a normalized noise equivalent absorption of $7.5\times 10^{-8}$ cm^−1^W/$\sqrt{\mathrm{Hz}}$. The work is meant to demonstrate the advantages of photothermal interferometry for NO_2_, such as miniaturization potential of the sensing volume, fast response times, and a robust setup.

## 2. Materials and Methods

### 2.1. Photothermal Interferometry

In this work, photothermal interferometry (PTI) is realized with a fiber-coupled Fabry–Pérot interferometer (FPI). Intensity modulation of the 450 nm excitation laser produces a thermal wave, with a temperature change directly proportional to the concentration of NO_2_ [22]. The thermal wave is heavily damped, and is therefore only observed close to the probe beam [7]. The local heating leads to a change in refractive index Δn, described by the Clausius–Mosotti equation [22], with ΔT temperature rise and $T_{abs}$ absolute temperature of the gas:

(1) $$\Delta n=-\left(n-1\right)\frac{\Delta T}{T_{abs}}.$$

Due to the constant gas flow through the cell, incremental heating of the gas sample due to the excitation laser can be neglected and constant absolute temperature of the gas can be assumed in our experiments. Hence, the detected change in refractive index is directly proportional to the NO_2_ concentration within the FPI cavity.

The FPI used for this work measures the intensity of the reflected probe laser. The reflected intensity $I_{r}$ is given by [23]

(2) $$I_{r}=I_{i}\left(1-\frac{1}{1+F\sin^{2}\left(\delta /2\right)}\right),$$

where $I_{i}$ is the incident intensity and *F* is the finesse of the mirrors. The phase shift δ depends on the refractive index in the cavity *n*, the distance between the mirrors *d*, and the wavelength λ as

(3) $$\delta =\frac{4\pi nd}{\lambda }.$$

Changes in *n* change the phase shift δ and, thus, the reflected intensity $I_{r}$.

### 2.2. Experimental Setup

**Figure 1:**
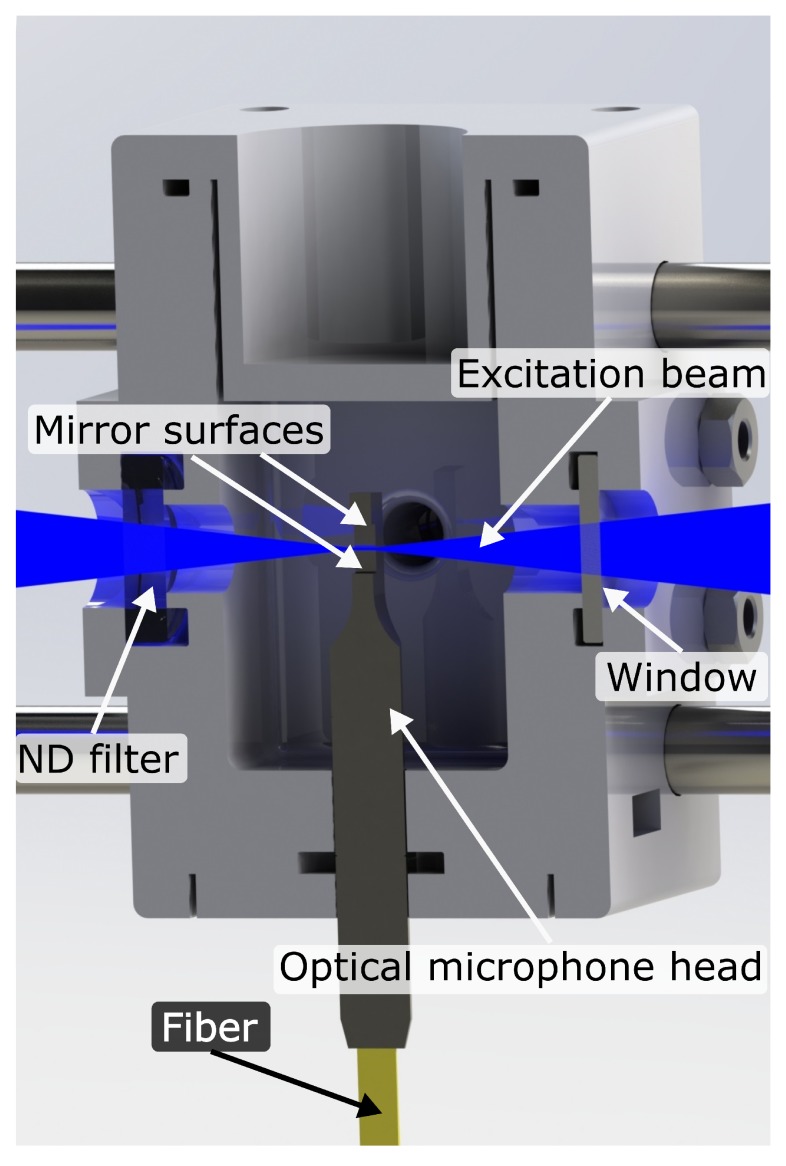
Cut through the 3D-printed cell, carrying the head of the optical microphone. Excitation laser beam is shown in blue. The probe beam is reflected within the microphone cavity between the top and bottom mirror surfaces.

For this work, a commercial optical microphone was used as detector, which consists of a fiber-coupled FPI cavity, machined as a rigid structure, which offers low sensitivity to mechanical vibrations [24]. The FPI is formed by a machined opening with semitransparent mirror surfaces, each approximately 1.5 mm × 1.5 mm in size, which are facing parallel to each other at a distance of approximately 3.3 mm. A 1550 nm probe laser of 1 mW optical power is reflected back and forth between the mirrors, and the reflected intensity is measured [24]. The probe laser is of approximately Gaussian shape, with 205 μm full width at half maximum within the cavity, and the reflectivity of the etalon mirrors is in the range of 0.6 [25]. The commercial microphone head comes with protective membranes covering the cavity, which were removed to allow for a free gas exchange and an overlap of the probe and excitation beams (cf. Figure 1).

The optical microphone is placed in a 3D-printed cell with a sample volume smaller than 9 cm${}^{3}$ (Figure 1). The cell was optimized with multiphysical simulations to suppress unwanted acoustic resonances. The beam of the excitation laser (blue) is focused through a window and horizontally centered to cross the probe laser of the optical microphone at the position of maximum intensity. A neutral density (ND) filter is mounted opposite the window to attenuate the excitation beam. A 40 mW continuous-wave optical power laser module (Laser Components GmbH, Olching, Germany: FLEXPOINT^®^ Dot Laser Module) with 450 nm wavelength serves as excitation laser. At this wavelength, NO_2_ yields high absorption with little cross-sensitivity to other gases. Also, this wavelength is above the photodissociation threshold [26].

**Figure 2:**
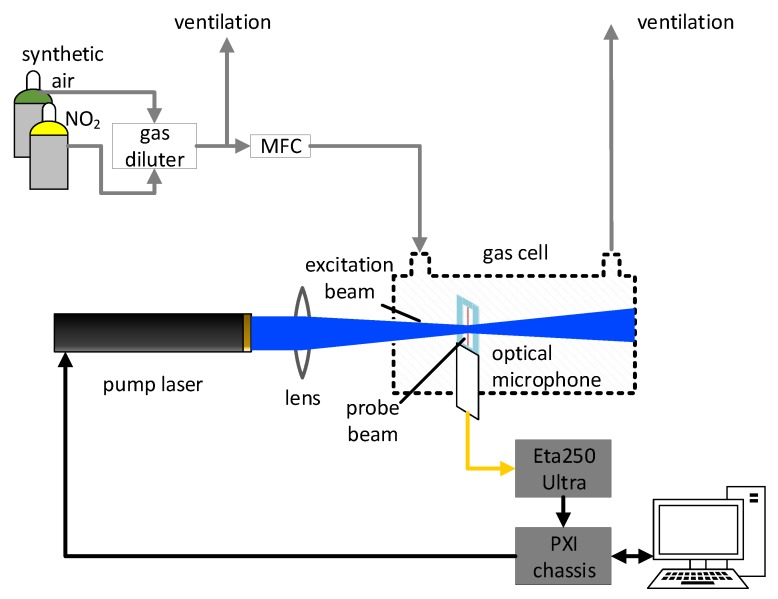
Schematic of the experimental setup for characterizing the photothermal interferometry setup with NO_2_. Gas mixtures are created with a gas diluter. The excitation beam is perpendicular to the probe beam and is focused and centered into the cavity of the optical microphone.

The experimental setup allows to study the sensor response for different concentrations of NO_2_, flow rates, and modulation frequencies. This is shown in Figure 2. Gas mixtures were produced with a temperature stabilized custom gas diluter based on binary weighted critical orifices [27], which offer low uncertainties over a broad range of dilution ratios. The NO_2_ gas cylinder contains a mixture of NO_2_ and synthetic air (Messer Austria GmbH, Gumpoldskirchen, Austria: 19.2 ppm NO_2_), which was further diluted with synthetic air (Messer Austria GmbH: Synthetic Air, Scientific). The flow rate to the PTI cell is controlled by a mass flow controller (MFC; Vögtlin, Aesch, Switzerland: Model GSC-B).

The signal from the optical microphone control unit (XARION Laser Acoustics GmbH, Vienna, Austria: Eta 250 Ultra—settings: Cutoff frequency 100 Hz, gain 20 dB) is recorded with a data acquisition card (National Instruments, Austin, TX, United States: Model PXI-6281) at 250 ksps and post-processed on a personal computer (PC). The same chassis carrying the PXI-6281 also houses a function generator (National Instruments: Model PXI-5402). The function generator provides the square-wave modulation signal (duty cycle 50%) for the excitation laser. The PTI signal is filtered with a digital lock-in amplifier, realized in LabVIEW code on a PC, with an integration time of 1 s. Due to the high sampling rate and limited buffer size of the DAQ card, data acquisition and modulation is stopped and restarted after each measurement to obtain a constant phase relation.

**Figure 3:**
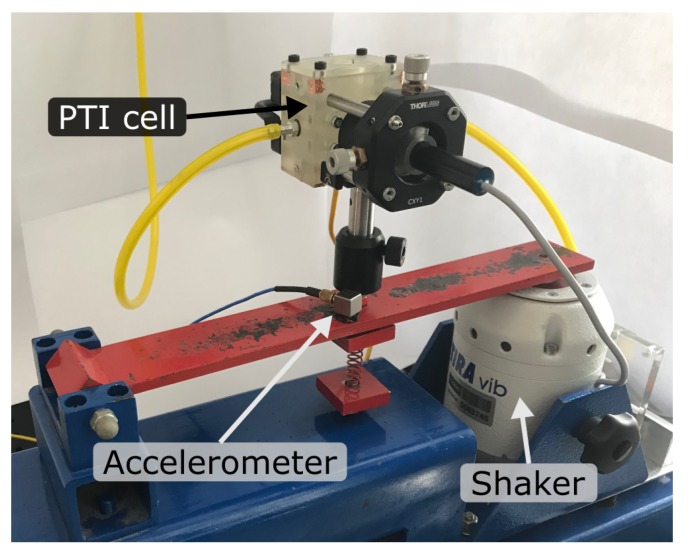
Photograph of the vibration test setup, showing the full photothermal interferometry (PTI) cell with gas lines and laser, mounted on an electrodynamic shaker. The piezoelectric accelerometer, measuring applied accelerations, is placed next to the cell mounting.

To investigate the sensitivity of the FPI and the signal to mechanical vibrations, the sensor was mounted on a platform connected to an electrodynamic shaker (TIRA GmbH, Schalkau, Germany: TIRAvib S502). Applied vertical accelerations and vibration spectral densities were measured with a piezoelectric accelerometer (PCB Piezotronics Inc., Depew, NY, United States: 333B30) placed next to the cell mounting, as shown in Figure 3. In this configuration the operating sensor was exposed to two different broadband quasi-random vibration distributions over the frequency range of 1 Hz–500 Hz—characteristic for highway truck vibration exposure [28]—to test mobile operation of the PTI sensor. The sensor was exposed to the acceleration spectra at different root mean square accelerations for several minutes and signal noise was recorded at multiple points in time. Additionally, the sensor was accelerated sinusoidally and maximum tolerable vertical accelerations (insignificantly increased signal noise) at a range of frequencies between 10 Hz and 300 Hz were determined. Due to the low anticipated effect on the noise level, the interferometer interrogation unit was not exposed to the vibrations.

## 3. Results

### 3.1. Influence of Flow Rate on Sensor Noise

**Figure 4:**
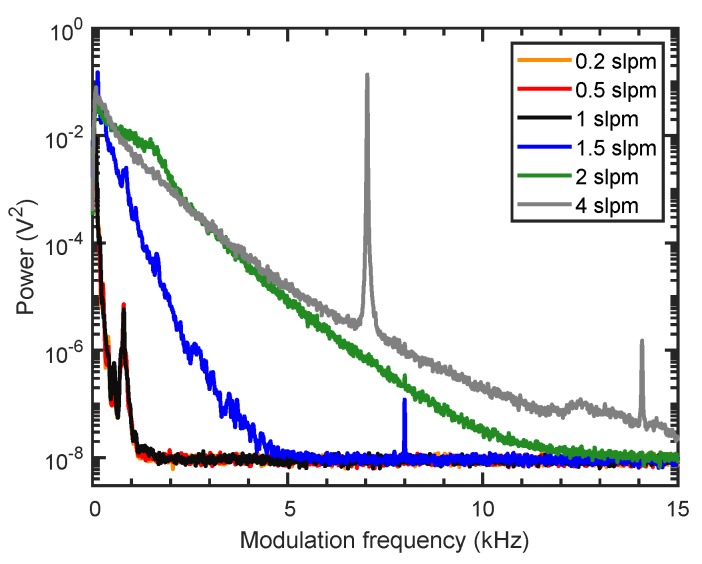
Welch spectra of the background noise without excitation laser at different flow rates. Welch spectra are calculated over 0.1 s windows and averaged over 9 s. The noise spectra of 0.2 slpm and 0.5 slpm are identical and covered by the spectrum at 1 slpm.

The selection of the flow rate is a balance of response time and detection limit, as higher flow rates offer faster gas exchange rates, but are associated to higher flow noise, which negatively effect the detection limit. Figure 4 shows the noise spectrum of the flow noise, measured by the optical microphone without the excitation laser being switched on. The noise spectrum was calculated as the Welch power spectral density estimate with a 0.5 s Hanning window. The flow rate was varied between 0.2 slpm and 4 slpm. Only a slight increase in noise is seen up to flow rates of 1 slpm, but higher flow rates significantly increase present $1/f^{\alpha }$ noise [29] and are accompanied by flow-rate-dependent tonal noise.

Due to the small cell volume of less than 9 cm${}^{3}$, a flow rate of 0.5 slpm with a nominal gas exchange rate of ≈1 s/cell volume was considered sufficient with a 1 s integration time, and was used in the subsequent measurements.

### 3.2. Selection of the Optimal Modulation Frequency

**Figure 5:**
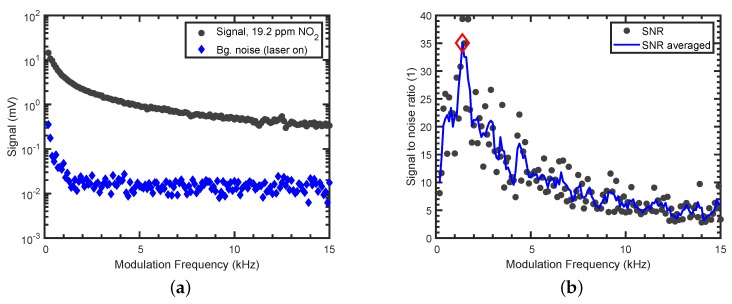
Noise investigations of the PTI sensor: (**a**) Signal with 19.2 ppm NO_2_ (black circles), background noise for different modulation frequencies with the laser switched on (blue diamonds), measured with lock-in amplifier. Background noise with the laser switched off equals the noise with the modulated laser switched on. (**b**) Signal-to-noise ratio as a function of the modulation frequency. The selected modulation frequency is marked with a red diamond.

From Figure 4, the additional presence of flow-rate-independent noise around 800 Hz is visible, and a higher modulation frequency should be chosen. However, as the photothermal signal is inversely proportional to the modulation frequency [22], a low modulation frequency is desired. Therefore, the signal with 19.2 ppm NO_2_ (black circles) and the background noise with synthetic air (blue diamonds) was recorded with the lock-in amplifier for different modulation frequencies (Figure 5a). The inverse signal strength of the photothermal signal can nicely be seen (black circles). To find the optimal modulation frequency, the signal-to-noise ratio (SNR) was calculated (black dots in Figure 5b). Due to the variation in the calculated SNR values, a moving average filter was applied (blue curve) and a modulation frequency of 1.4 kHz, in the region where best results were achieved, was selected.

### 3.3. Limit of Detection and Long Term Stability

**Figure 6:**
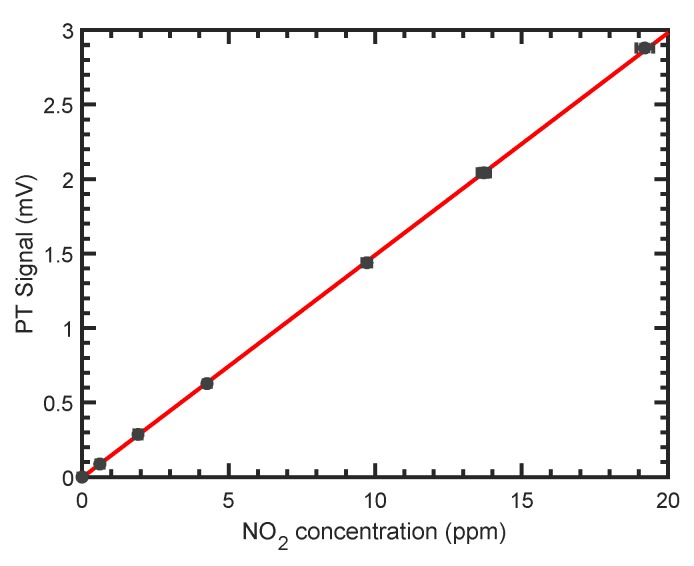
Linear fit (red) of the background-corrected photothermal signal as a function of the NO_2_ concentration. Error bars of the photothermal signals are the standard deviation relative to the mean. Error bars of the concentrations are too small to be visible on this scale.

The linearity of the PTI sensor was confirmed by applying concentrations ranging from 606 ppb to 19.2 ppm NO_2_ to the sensor. The PTI signal, as a function of applied NO_2_ concentration, is shown in Figure 6. Each data point was averaged for approximately 40 values, i.e., 40 s. All signals are background-corrected with respect to their phase. The sensitivity was determined to be $(0.149\pm 0.002)\;mV/\mathrm{ppm}{}^{-1}$ from a weighted linear regression. The coefficient of determination for the fit is $R^{2}=0.999$.

**Figure 7:**
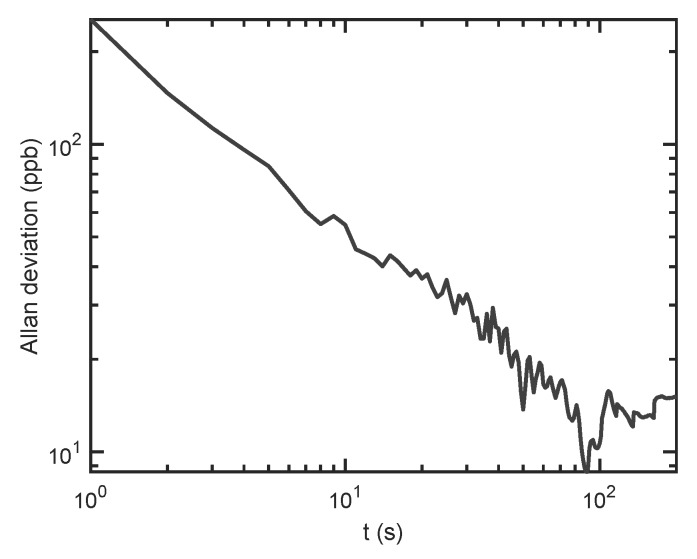
Allan deviation of the photothermal signal in units of NO_2_ concentration as a function of averaging time. Allan deviation was calculated with MATLAB.

Long-term stability was investigated by calculating the Allan deviation for the signal at constant flow of synthetic air. The corresponding plot is shown in Figure 7. Even though the sensor was mounted on an optical table without vibration isolation, no interferences from mechanical vibrations were observed, due to the rigid structure of the FPI.

From Figure 7, it can be seen that the 1σ detection limit is 348 ppb for 1 s averaging time. The detection limit can be further improved to 75 ppb (10 s) and 26 ppb (100 s), by using longer averaging times. The increase in standard deviation after approximately 200 s, and the therefore limited maximum integration time, stems from the laser module, which is not temperature stabilized (cf. Appendix A). This, however, could be easily improved by changing to a temperature stabilized laser. The optical microphone itself uses a feedback current to stabilize the wavelength of the probe laser to maintain a steady operating point, i.e., compensating for slow temperature and pressure changes [24].

The normalized noise equivalent absorption was calculated by assuming a Gaussian wavelength distribution around 450 nm with FWHM of 0.5 nm, and using the corresponding absorption coefficient from the HITRAN database [30]. For 1 s integration time of the lock-in amplifier, this corresponds to an NNEA of $7.5\times 10^{-8}$ cm^−1^W/$\sqrt{\mathrm{Hz}}$.

**Figure 8:**
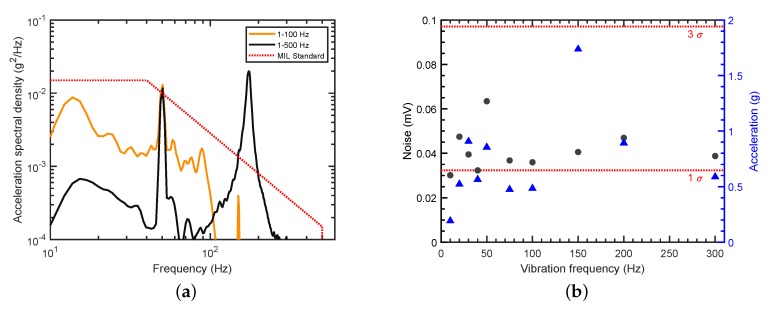
Vibration analysis: (**a**) Broadband acceleration spectral densities applied to the measurement setup during operation (yellow and black curves). Military standard vibration schedule for highway truck vertical vibration exposure (MIL-STD-810H, Method 514.8C-I [28]; red dotted line). (**b**) Measured signal noise (black circles) at the applied sinusoidal vertical peak accelerations (blue triangles) for zero air. Horizontal dotted lines mark 1σ and 3σ noise levels.

Low sensitivity of the PTI sensor to mechanical vibrations is essential for mobile applications and is usually hard to achieve for interferometric setups. Results of the vibration analysis, however, suggest low sensitivity of the proposed interferometric sensor concept, due to the rigid structure of the FP cavity. Figure 8a shows the applied acceleration spectral densities in the frequency range of interest and, for comparison purposes, a military standard vibration schedule for highway truck vertical vibration exposure, often used for commercial product testing (MIL-STD-810H, Method 514.8C-I [28]). The peak visible at 50 Hz in both spectra is noise at the power line frequency, amplified by the accelerometer amplifier, and has to be disregarded from the acceleration spectrum. Although a strong mechanical resonance of the setup is excited near 150 Hz for vibrations up to 500 Hz (black curve), the measured signal noise level only increases marginally from 30 μV to 40 μV. During application of the low-frequency vibration spectrum with components up to 100 Hz (yellow curve), no changes in the measured noise level were observed and the noise remained at the background level.

Achieved peak accelerations for sinusoidal vibrations are plotted in Figure 8b together with the measured noise. It can be seen that, for frequencies between 20 Hz and 300 Hz, the PTI sensor was exposed to accelerations at or above 0.5 g up to 1.7 g, with the noise level still well within the 3σ noise band. At 10 Hz, the large motion amplitude of the electrodynamic shaker was causing a repeated mechanical impulse to the PTI setup, and applied peak accelerations were reduced to prevent impulse excitation.

### 3.4. Response Time

**Figure 9:**
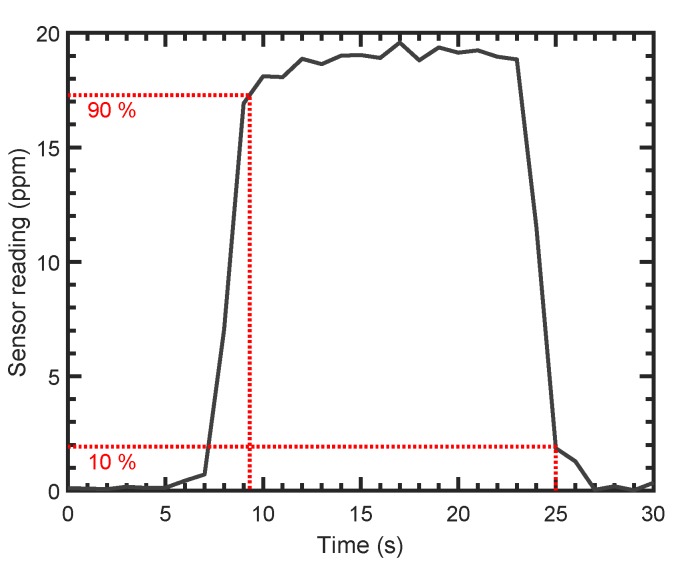
Sensor step response for a step from zero air to a concentration of 19.2 ppm NO_2_ and back at 0.5 slpm and 1 s integration time. Response time to 90% ($\tau_{90}$) and recovery time to 10% ($\tau_{10}$) signal level are below 3 s and 2 s, respectively.

As short response times are critical for a wide variety of applications, the response of the proposed PTI sensor to steps in concentration was investigated. Repeated steps from synthetic air (zero concentration) to concentrations of 19.2 ppm NO_2_ at 0.5 slpm and 1 s integration time revealed reproducible response times to 90% signal level ($\tau_{90}$) below 3 s; and recovery times to 10% signal level ($\tau_{10}$) below 2 s. An exemplary response curve is shown in Figure 9.

## 4. Discussion and Conclusions

The presented sensor concept, using a compact fiber-coupled Fabry–Pérot interferometer for photothermal spectroscopy, offers a reliable sensing scheme for NO_2_ with high spectral selectivity and sensitivity. The NNEA was determined to be $7.5\times 10^{-8}$ cm^−1^W/$\sqrt{\mathrm{Hz}}$, which is lower than previous PTI implementations [15,16], but can still be improved in future realizations, e.g., by applying a balanced detection scheme [17].

QEPAS implementations reach slightly better NNEAs (e.g., $2.5\times 10^{-8}$ cm^−1^W/$\sqrt{\mathrm{Hz}}$ [10], $4.2\times 10^{-9}$ cm^−1^W/$\sqrt{\mathrm{Hz}}$ [13]) and, compared to conventional PAS implementations, the NNEA is up to a factor 100 worse (7.0×10−10 cm^−1^W/$\sqrt{\mathrm{Hz}}$ [10]). However, the proposed PTI sensor approach offers the possibility for a much smaller detection volume, capable of faster response times and higher miniaturization potential.

The developed, non-optimized cell has a volume smaller than 9 cm${}^{3}$, for which an integration time of 1 s combined with a flow rate of 0.5 slpm proved to provide a good balance of response time and detection limit. Even though the given cell geometry comprises poorly flushed dead volumes, a response time of $\tau_{90}=3s$ and a recovery time of $\tau_{10}=2s$ were achieved. For applications requiring faster response times, a combination of smaller integration time and higher flow rate can be easily realized. Due to the small size of the optical microphone, the cell volume could be ultimately decreased to the dimensions of the FPI cavity, which is $1.5mm\times 1.5mm\times 3.3mm\approx 7.5mm^{3}$, without major drawbacks. This is highly advantageous when compared to microstructured hollow-core fiber-based PTI approaches, where long fibers are needed to reach comparable NNEAs. To fully demonstrate the miniaturization potential of the presented method, future research should focus on the downscaling of the cell down to the FPI cavity volume.

On the other hand, applications like environmental monitoring require lower detection limits, at averaging times of up to one hour [5]. In this case, a stabilized laser source could be used, which would enable longer averaging times to improve the detection limit. Additionally, higher laser power could be used to improve the detection limit, as the photothermal signal scales directly with the laser power.

A low sensitivity of the PTI sensor to mechanical vibrations was demonstrated with broadband vibrations in a frequency range similar to vehicular vibration profiles. Although the applied vibration power was below the specified root mean square acceleration of $g_{rms}=1.04g$ in the cited military vibration test standard, future commercial application possibilities in mobile gas sensing should be realizable with minor improvements in setup stability. This is underlined by the fact that, for sinusoidal vibrations, peak accelerations of 1.7 g could be applied to the described setup, without significantly increasing signal noise.

Although an expensive lab grade optical microphone was used for the proof of principle experiments, chip-level miniaturization of the sensor is possible, offering interesting potential for large-scale production of the sensor. Possible fields of application include exhaust gas and emission measurements. Furthermore, measurement of different gases can easily be achieved by using excitation lasers of different wavelengths.

## Figures and Tables

**Figure A1:**
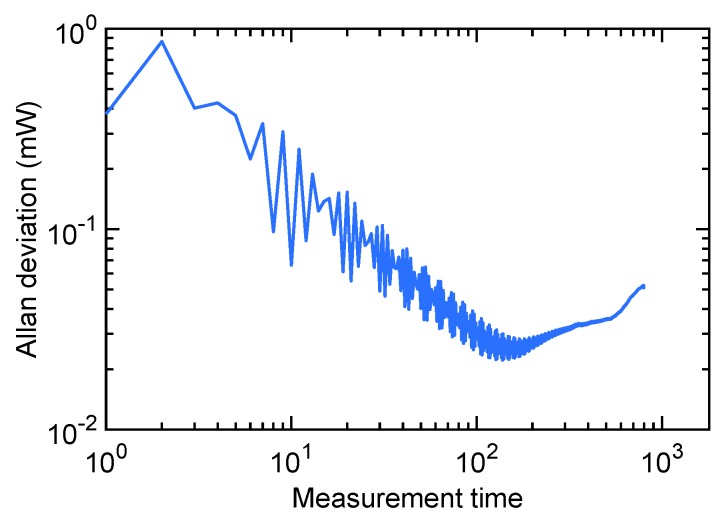
Allan deviation of the laser power as a function of the averaging time.

## Abbreviations

The following abbreviations are used in this manuscript:

PTI	photothermal interferometry
PAS	photoacoustic spectroscopy
QEPAS	quartz-enhanced photoacoustic spectroscopy
ksps	kilosamples per second
NNEA	noise normalized equivalent absorption

## Appendix A. Drift of the Laser Power

To investigate whether drift is dominated by fluctuations of the laser power, an Allan deviation analysis was carried out. As can be seen in Figure A1, a drift of the laserpower is appearing at the same timescale as the drift of the FPI sensor (around 200 s), which indicates that drift is caused by fluctuations of the laser power.
